# Gossypiboma Resection after Eight Years in a Patient with Rheumatoid Arthritis and Diabetes

**DOI:** 10.1155/2017/3239093

**Published:** 2017-10-03

**Authors:** Kenley Unruh, Hsien Sing Sam Hsieh

**Affiliations:** ^1^University of Washington School of Medicine, 1959 NE Pacific St, Seattle, WA 98195, USA; ^2^Coulee Medical Center, 411 Fortuyn Road, Grand Coulee, WA 99133, USA

## Abstract

Gossypiboma is the term used to refer to a mass formed by surgical material left in the body cavity after surgery. We present the case of a middle-aged woman with a history of rheumatoid arthritis controlled with corticosteroids and biologic therapies, uncontrolled type II diabetes mellitus, and cesarean section with postoperative bleeding eight years earlier, who presents with right lower quadrant abdominal pain and is found to have a gossypiboma from her previous operation. A subsequent operation is undertaken to remove the gossypiboma. After the procedure, our patient's diabetes and chronic back pain greatly improve, raising the question of gossypiboma's role in these diseases. A review of our patient's records found that a correct sponge count was recorded after her cesarean section, raising questions about the operating room policies regarding surgical counts, the presence of falsely correct counts, and the need for postoperative plain films in procedures with an increased risk of a retained object. Our patient's presentation eight years after the inciting surgery raises questions about the involvement her immunosuppressive therapy may have had in cloaking the gossypiboma. Our case also raises the question of surgical culpability, including the ethical and legal considerations for apology from the culpable surgeon.

## 1. Introduction

Gossypiboma is the term used to refer to a mass formed by surgical material left in the body cavity after surgery. This retained material is usually textile, most commonly in the form of a surgical sponge [1]. It is an unusual occurrence, with 1 : 1000 to 1 : 1500 surgical cases resulting in a retained foreign body, but the severe consequences of infection, a second operation to remove the material, and possible legal action warrant interventions in the operating room to prevent such an occurrence [2]. Not all surgical cases have the same rate of retained surgical material, with abdominal operations resulting in the highest occurrence of this unfortunate outcome [3]. We present the case of a middle-aged woman with a history of rheumatoid arthritis, type II diabetes mellitus, and cesarean section with postoperative bleeding eight years previously, who presents with right lower quadrant abdominal pain and is found to have a gossypiboma from her previous operation.

## 2. Case Report

**Figure 1:**
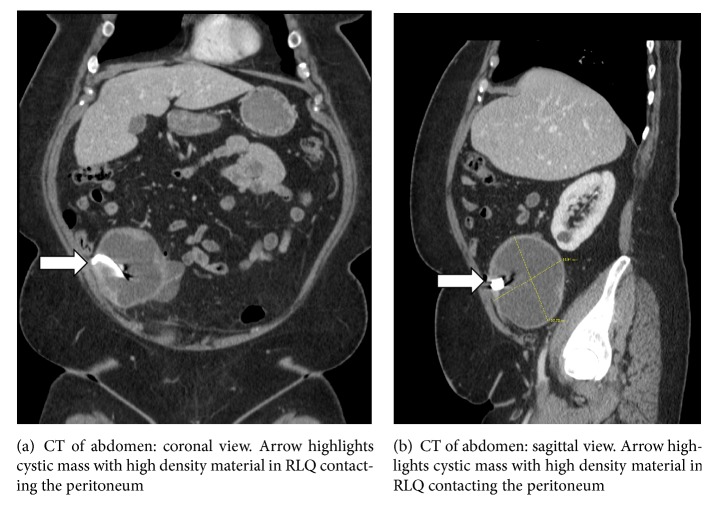


A 46-year-old female with a history of rheumatoid arthritis controlled with corticosteroids and biologic therapies, uncontrolled type II diabetes, and a history of a cesarean section with postoperative bleeding eight years previously presents to the emergency department with “achy pressure” in her right lower quadrant worsening over the past seven days. The patient states the pain worsens with moving or lying flat and has been radiating to her right leg. She has also been feeling more bloated over the past week, though eating does not seem to affect the pain. The patient's last bowel movement was 1 day ago. The patient endorses chronic back pain but denies any groin pain, melena, hematochezia, fever, chills, nausea, vomiting, or diarrhea. Her menstrual periods are very irregular, with her last menstrual period occurring “months ago.” Physical exam reveals temperature = 36.4 C (97.5 F), blood pressure = 132/78 mmHg, and pulse = 69 beats/min, with right lower quadrant (RLQ) tenderness with rebound tenderness and positive bowel sounds. Laboratory values in the emergency department are significant only for elevated glucose, with no signs of infection or pregnancy (Table 1). Urinalysis is performed, revealing only high urine glucose, moderate hematuria, and no visible bacteria. Computed tomography (CT) of the abdomen and pelvis with contrast shows a large, complex, cystic mass interposed between the appendix and right ovary, measuring 11.9 × 9.4 × 11.4 cm. The mass contains a high density, ribbon-like material consistent with a laparotomy sponge marker, but ovarian origin of the mass cannot be excluded (Figures 1(a) and 1(b)). The patient is placed on an insulin drip and prophylactic antibiotics and kept overnight for next-day diagnostic laparoscopy to investigate the mass.

**Figure 2:**
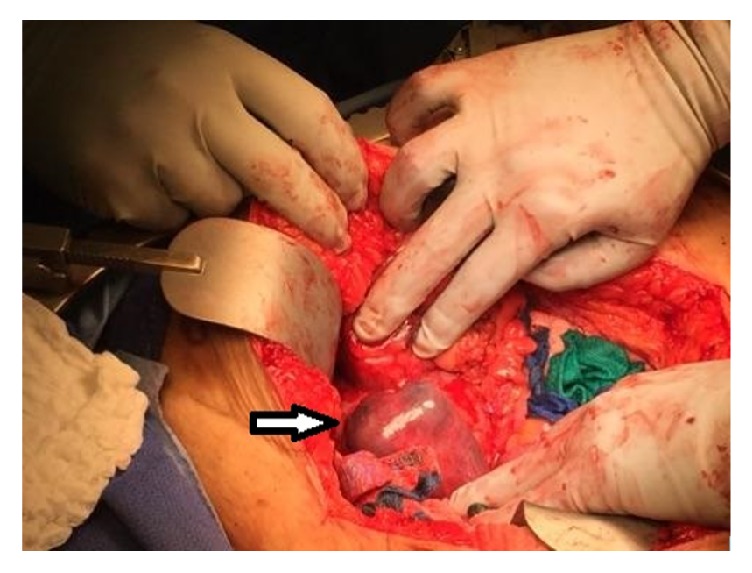
Intraoperative photo. Arrow highlights cystic mass in RLQ.

**Figure 3:**
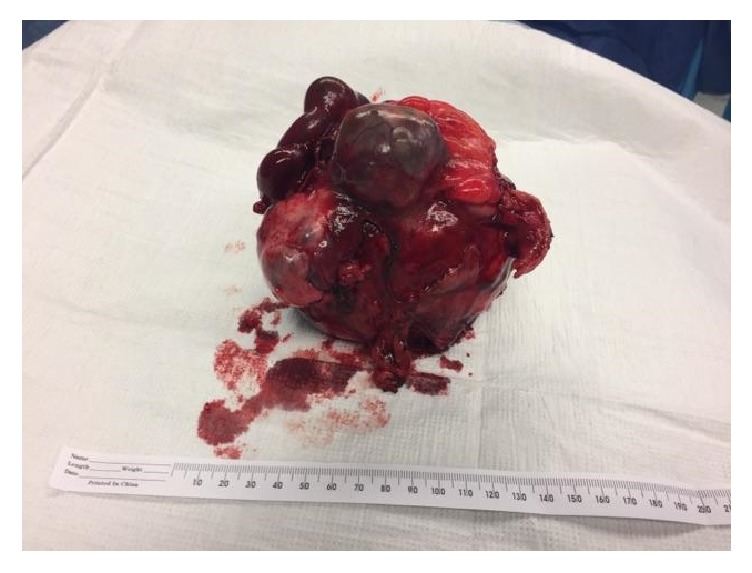
Postoperative photo of intact gossypiboma specimen with attached right ovary and portion of small bowel.

**Figure 4:**
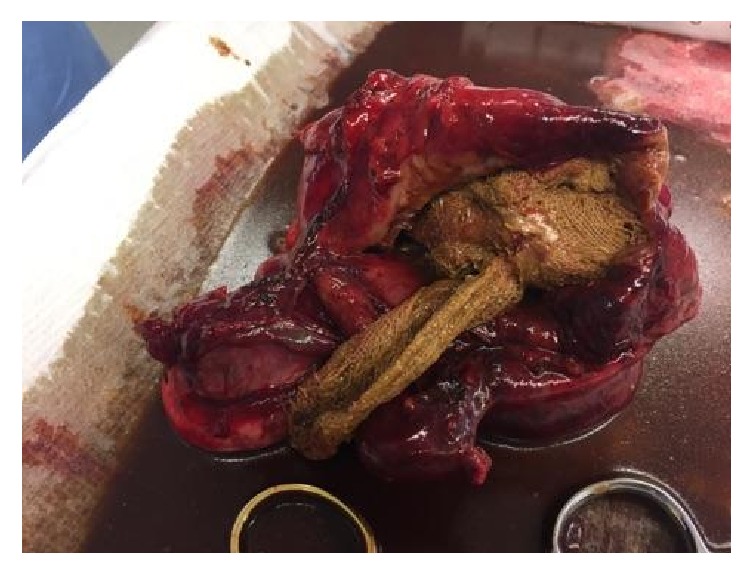
Postoperative photo of dissected gossypiboma specimen showing the retained laparotomy sponge from our patient's previous operation.

Diagnostic laparoscopy reveals a cystic mass with dense adhesion of surrounding organs. At this point, it is still indeterminate whether the mass is a laparotomy sponge or it is of ovarian origin. The procedure is converted to open. This reveals a large, thick, cystic mass in the RLQ densely adherent to a segment of small bowel, cecum (including the appendix), right fallopian tube, right ovary, and the retroperitoneal wall (Figure 2). An en bloc resection is performed, with a subsequent right salpingo-oophorectomy, partial jejunectomy, and partial cecectomy with appendectomy. The cystic mass is then removed from the abdomen and dark, green fluid is aspirated from it. The mass is then dissected, revealing a laparotomy sponge encapsulated in the mass (Figures 3 and 4). The entire mass and sponge are sent in formalin for pathologic review.

Postoperatively, the patient further reveals that after her cesarean section she had not experienced any postoperative pain until her current presentation, despite the excessive bleeding and emergent closure during the cesarean section. By postoperative day four, the patient is able to spontaneously void both bladder and bowel, after which her drain is removed and she is discharged on a home insulin regimen. She is encouraged to follow up with her rheumatologist regarding restarting her rheumatoid arthritis therapy.

A review of records from the patient's 2009 cesarean section reveals that the operation was performed emergently due to concerns for preeclampsia. During the operation, the patient's uterus was atonic and hemorrhaging after the child's delivery, resulting in 1500 mL of blood loss. The patient required doses of both carboprost tromethamine (Hemabate) and methylergometrine (Methergine) to increase uterine tone and control bleeding. The patient was quickly closed with correct second and final sponge/needle counts. Postoperatively, the patient was given two units of blood and recovered well, with minimal serosanguineous drainage from her incision and some incisional discomfort. Follow-up appointments over the subsequent weeks reveal some fullness superior to the incision right of midline. This is attributed to an underlying seroma, which eventually resolves.

After her mass resection, the patient follows up multiple times over the following six weeks, with improvement in her blood sugar control as well as resolution of her chronic lower back pain that had been present since her cesarean section.

## 3. Discussion

Gossypiboma is the term used to refer to a mass formed by surgical material left in the body after surgery. Risk factors for retained surgical material include emergency surgery, high patient body mass index (BMI, calculated as body mass divided by the square of body height), unplanned changes in surgical procedure, intraoperative complications, long operation duration, inexperienced staff, incorrect sponge count, shift changes of surgical team, and involvement of more than one surgical team in the operation, with only the first three risk factors being shown to be statistically significant in multivariate analysis [4]. Our patient and her cesarean section operation included three of these risk factors: emergency surgery, high patient BMI (37 at the time of her cesarean section), and intraoperative complications, which help to explain her unfortunate complication. A case could be made that our patient's BMI of 37 is not entirely accurate as its high value at the time of her operation can be attributed to her pregnancy rather than purely an increase in body mass. It is unclear if the cause of an increased BMI changes a patient's risk of gossypiboma (pregnancy being the cause in our patient's case) or if pregnancy itself is a risk factor for gossypiboma, but these questions demand further consideration when assessing a patient's risk for gossypiboma.

Even with these risk factors, the retention of a laparotomy sponge in our patient was due to a combination of human error and inadequate policy regarding proper surgical material accounting. In accordance with the Joint Commission on the Accreditation of Healthcare Organization (JCAHO) classification of retained surgical material as a reportable sentinel event, a root-cause analysis (RCA) was performed for this incident [5]. The RCA found that the operative report by the surgeon reported the sponge and instrument counts to be correct; but during the operation, the sponge counts before, during, and after the procedure were signed off by the circulating nurse and were not specified to be correct or incorrect on the circulating nurse's worksheet. This discrepancy raises the question of the actual outcome of the sponge count at the time of the operation. A review of the “Accountability for Sponges, Sharps, and Instruments” policy in place at the operating institution at the time of the operation specified that if an incorrect count was performed, a thorough search for the missing sponge should be conducted. If that search were unfruitful, an intraoperative radiograph should be performed to rule out its location within the patient (this policy was found to be in accordance with the recommended practices for sponge, sharp, and instrument counts by the Association of Perioperative Registered Nurses) [6]. Because this intraoperative radiograph was not performed on our patient, we can assume the count was reported as correct during the operation but falsely so.

This situation highlights the deception of a correct surgical count. Multiple studies have found the majority of cases with retained surgical material to have falsely correct counts [4, 7]. This fact, and its presence in our patient's case, highlights the need for an additional policy of performing intraoperative radiographs regardless of correct counts for cases including one or more of the risk factors for retained surgical material (as highlighted above) [4, 8, 9]. Some have suggested adding an intraoperative or postoperative radiograph for all procedures, but a cost-effectiveness report found that such a “universal” radiograph strategy is prohibitive due to an estimated cost of >$1.3 million per retained object prevented [9]. The addition of a selective radiograph policy would help account for the risk of a falsely correct count without such a high cost.

The resolution of our patient's back pain and the marked improvement of her diabetes status after resection of the gossypiboma highlights the potential connection between retained surgical material and other illnesses. The signs of local inflammation seen during our resection, as well as the marked improvement upon resection of the gossypiboma, make this mass a contributing factor (if not the most likely cause) of our patient's back pain. There is also a known correlation between inflammation and systemic diseases such as type II diabetes [10–13]. A foreign object that had caused enough inflammation to form its own cystic structure and erode into bowel, viscera, and our patient's right ovary could also create a significant systemic immune response, thereby increasing the levels of systemic inflammatory cytokines, which are thought to be a contributing factor to diabetes [13]. Although there is a known correlation between systemic inflammation and type II diabetes, no causation between the two has been proven. In our patient's case, the marked improvement in our patient's blood sugar control after resection of the gossypiboma may point toward an inflammatory component of her diabetes. This improvement in both our patient's diabetes and back pain status after gossypiboma resection highlights the need for a low index of suspicion for retained surgical material for clinicians whose patients present with complaints of pain or masses postoperatively or for patients whose systemic diseases worsen after their procedure.

When a surgical object is retained after an operation, the body can mount two types of reactions to that object: an acute exudative response usually resulting in early symptoms or a chronic, aseptic, fibrinous response that can encapsulate the retained surgical object and create a cystic mass that either is asymptomatic or presents with minor symptoms [14]. This “gossypiboma” can adhere to adjacent structures, including bowel, ovary, and peritoneum, and will eventually attempt to extrude itself from the body cavity along the path of least resistance, usually along a sinus tract or into a hollow viscus [15]. When these gossypibomas develop, the average time for diagnosis of the mass is around five years [16]. Our patient presented eight years after her inciting operation, raising the question of the role her use of immunosuppressive medications played in the formation and presentation of the gossypiboma. A lack of immune response due to our patient's use of corticosteroids and biologic therapies could have resulted in subclinical symptoms from her retained laparotomy pad, as well as a slowing of the formation and migration of the gossypiboma. This highlights the concern that patients who undergo surgery while using immunosuppressive therapy may not have obvious presentations if surgical material were to be retained, instead of presenting with minimal or insidious symptoms that require a low index of suspicion to be noticed and pursued.

Another interesting aspect of our case is the ethical consideration of the physician who performed our patient's cesarean section. This physician approached our patient prior to her diagnostic laparotomy to apologize for the possibility that what she was experiencing was due to a mistake on his part. Though the diagnosis of gossypiboma was not confirmed, this physician felt it was his responsibility to visit the patient and make a “prophylactic apology,” emphasizing he had never had an outcome like this before and he “felt horrible” for what had happened to our patient. During this interaction, the patient was very understanding of the situation, especially the emergent nature of the cesarean section, and thanked him for coming to see her before her operation. Not only does this disclosure and apology by the original operating physician comply with the requirements of the JCAHO for accreditation, the American Medical Association's code of ethics, and the American College of Physicians' ethics manual, but also it emphasizes the need for clear communication and empathy regarding adverse outcomes with patients [17–19]. Using open communication with patients has been shown to decrease the likelihood of subsequent legal action against the physician and can also reduce the amount of compensation requested by patients if they are to litigate [20, 21]. Though only a small proportion of patients who are injured due to adverse medical outcomes sue the offending physician and/or hospital, clear communication between the physician and patient is still imperative regarding adverse outcomes. This is to help maintain the doctor-patient relationship and for the mental health of both the physician and patient [22, 23].

Our case raises many questions regarding incidents of retained surgical material, including policies and procedures to limit its occurrence, the inflammatory role a retained object may have on a patient's systemic diseases, the effects of immunosuppressive treatments on the presentation of retained surgical material, and the ethical and legal considerations regarding disclosing such an event to the patient. Hopefully, our experience will set the groundwork for increased prevention and further study of this persistent but preventable negative outcome.

## Figures and Tables

**Table 1:** Laboratory values for emergency department.

	Emergency department	Normal ranges
Glucose	331 mg/dL	65–99 mg/dL
Sodium	136 mmol/L	137–145 mmol/L
Leukocyte count	10.2 k/uL	4.0–11.0 k/uL
Neutrophil count	7.3 k/uL	2.0–7.3 k/uL
Lymphocyte count	2.49 k/uL	1.0–3.4 k/uL
Globulin	3.8 g/dL	2.4–3.5 g/dL
Hemoglobin	17.0 g/dL	11.6–15.5 g/dL
Platelet count	170 k/uL	150–400 k/uL
Pregnancy test	Negative	
